# Separate roles for Med12 and Wnt signaling in regulation of oxytocin expression

**DOI:** 10.1242/bio.031229

**Published:** 2018-03-12

**Authors:** Emma D. Spikol, Eric Glasgow

**Affiliations:** Department of Oncology, Georgetown University, 4000 Reservoir Rd., Washington, DC 20057, USA

**Keywords:** Zebrafish, Brain development, Hypothalamus, Neuroendocrine, Transcriptional regulation

## Abstract

Transcriptional control of oxytocinergic cell development influences social, sexual, and appetite related behaviors and is implicated in disorders such as autism and Prader-Willi syndrome. Mediator 12 (Med12) is a transcriptional coactivator required for multiple facets of brain development including subsets of serotonergic and dopaminergic neurons. We surveyed hormone gene expression within the hypothalamo-pituitary axis of *med12* mutant zebrafish embryos with a focus on *oxytocin* (*oxt*) expression. Some transcripts, such as *oxt*, vasopressin (*avp*) and *corticotrophin releasing hormone* (*crh*) are undetectable in the *med12* mutant, while others are upregulated or downregulated to varying degrees. In *med12* mutants, the expression patterns of upstream transcriptional regulators of oxytocinergic cell development remain largely intact in the pre-optic area, suggesting a more direct influence of Med12 on *oxt* expression. We show that Med12 is required for Wnt signaling in zebrafish. However, *oxt* expression is unaffected in Wnt-inhibited embryos indicating independence of Wnt signaling. In fact, overactive Wnt signaling inhibits *oxt* expression, and we identify a Wnt-sensitive period starting at 24 h post fertilization (hpf). Thus, Med12 and repression of Wnt signaling display critical but unrelated roles in regulating *oxt* expression.

## Abstract (precis)

**Summary:** Mediator 12, a transcriptional coactivator, greatly enhances Wnt signaling in the developing embryo. Separate from its role in Wnt signaling, Mediator 12 is required for *oxytocin* expression.

## INTRODUCTION

The neuroendocrine system connects the brain and body to control homeostasis, mood, metabolism and sexual behaviors. These functions are mainly regulated by the hypothalamus, which is composed of distinct cell populations that integrate sensory signals and release neuropeptides that act on other neuron populations and circulate in the bloodstream. Neurons from the hypothalamus project throughout the brain and to the anterior and posterior pituitary, forming the hypothalamo-pituitary axis. Disruptions in the development of the hypothalamus have been linked to depression, chronic stress, autism and obesity. Thus, efforts are being made to understand how transcription factors and secreted growth factors coordinate the development of this brain region (Biran et al., 2015; Pearson and Placzek, 2013). Here, we report previously unrecognized roles for the transcriptional co-activator Mediator 12 (Med12) and the Wnt signaling pathway in regulating expression of the hypothalamic neuropeptide oxytocin.

The oxytocin system has generated increasing interest in recent decades due to the role this neuropeptide might play in regulating social behavior and mental health. Oxytocin is traditionally known for facilitating birth and milk let-down in mammals, and is thought to control reproductive behavior, social bonding and attachment (Kumsta and Heinrichs, 2013). In fact, several studies report associations between SNPs in the oxytocin receptor (OXTR) gene, and autism spectrum disorder (ASD) (Ebstein et al., 2012; Wu et al., 2005). Other reports have proposed roles for the oxytocin system in regulating stress, mood and appetite (McQuaid et al., 2014; Sabatier et al., 2013). In zebrafish *oxt* is expressed in the pre-optic area (PO), which is likely the evolutionary precursor to the mammalian oxytocinergic neuron clusters, the paraventricular nucleus (PVN) and the supraoptic nucleus (SON) (Biran et al., 2015; Herget et al., 2014). The anatomical features of the hypothalamo-pituitary axis are remarkably conserved between zebrafish and mammals. In both cases, magnocellular neurons produce oxytocin, which travels to the neurohypophysis by axonal projections, where it is released into systemic circulation (Löhr and Hammerschmidt, 2011; Eaton et al., 2008). Importantly, oxytocinergic neurons also project throughout the central nervous system where oxytocin acts as a neuromodulator. Complex projections of oxytocinergic neurons to the tectum, midbrain and hypothalamus have been visualized in larval zebrafish (Herget et al., 2017; Coffey et al., 2013).

Reduction of oxytocin expression can have detrimental effects on human health, as evidenced by the neurobehavioral disorder Prader-Willi Syndrome (PWS), which is characterized by dysregulated social behavior and insatiable appetite. The basis for this disorder is the loss of paternally expressed genes in the 15q11-q13 region, which are imprinted and therefore not expressed from the maternal chromosome. Affected genes include ubiquitin ligases, RNA processing regulators and SnoRNAs. Although oxytocin expression is reduced in PWS, how the genetic deletions that cause the disorder relate to this gene expression change is an open question (Spikol et al., 2016). Of interest are the POU homeobox gene POU3F2, the bHLH-PAS gene SIM1, and the paired class homeobox gene OTP, of which the orthologous genes are required for oxytocin expression in zebrafish and mouse (Eaton and Glasgow, 2006, 2007; Kasher et al., 2016; Acampora et al., 1999; Blechman et al., 2007; Fernandes et al., 2013; Löhr et al., 2009; Michaud et al., 1998; Nakai et al., 1995; Schonemann et al., 1995; Wang and Lufkin, 2000). In fact, monogenic syndromes with similar characteristics to PWS resulting from loss of SIM1 or POU3F2 have been characterized (Izumi et al., 2013; Kasher et al., 2016). However, considering the complex roles of genes that are lost in PWS, it is likely that other transcriptional modulators and signaling pathways that regulate hypothalamic development are perturbed in the disorder, hinting at as-yet-unknown mechanisms that control oxytocin expression.

In the developing brain, spatially localized signals control differentiation of progenitor populations into specific cell types. The transcriptional co-activator Mediator 12 (Med12) was proposed to act as a molecular switch in this process, regulating cell fate decisions for serotonergic, noradrenergic and dopaminergic neuronal subtypes without affecting global neuron number (Wang et al., 2006). Med12 is a subunit of the CDK8 regulatory module of the Mediator complex, which facilitates the interaction between gene-specific transcription factors and general transcription machinery to activate transcription (Malik and Roeder, 2010). In humans, mutations in MED12 cause two, rare, intellectual disability syndromes called FG syndrome and Lujan-Fryns (LF) syndrome, while in-frame insertions and deletions in MED12 are associated with a 1.8-fold increased risk of schizophrenia (Wu et al., 2014; Risheg et al., 2007; Schwartz et al., 2007; DeLisi et al., 2000). The mechanisms by which Med12 controls cell fate determination are not well understood. One possibility is that Med12 is required for transcriptional response to signaling pathways at work during development. Reports from cell culture and mouse studies indicate that Med12 is largely required for β-catenin to activate Wnt-dependent transcription (Kim et al., 2006; Rocha et al., 2010). Interestingly, Wnt signaling is thought to be involved in specifying hypothalamic versus floorplate identity, as well as stimulation of neurogenesis in the hypothalamus (Machluf et al., 2011; Xie and Dorsky, 2017; Kapsimali et al., 2004; Lee et al., 2006).

Here, we asked whether Med12 plays a role in the development of oxytocinergic neuron clusters, since it is partly required for serotonergic and catecholaminergic populations. We found that oxytocin expression is undetectable in the *med12^y82/y82^* mutant and characterized the effect of the *med12* mutation on neurosecretory and hormone-producing cells of the hypothalamus and pituitary. Next, we asked whether Med12 is required for Wnt-dependent transcription in zebrafish. We found that while Med12 is required for Wnt signaling in zebrafish, inhibition of Wnt signaling does not lead to a reduction in oxytocin expression. To assess the effect of overactive Wnt signaling, we used the *apc ^hu745/hu745^* mutant, which leads to accumulation of β-catenin and constitutive activation of the Wnt pathway (Hurlstone et al., 2003). Our results indicate that Wnt signaling must be repressed, starting around 24 h post fertilization (hpf), when oxytocin cells are likely beginning to express oxytocin, which is the defining step in the differentiation of oxytocin cells (Unger and Glasgow, 2003). The data presented here enhance current understanding of how the transcriptional activator Med12 and the Wnt signaling pathway control the fate decisions of specific neuronal subtypes, and suggest that dysregulated Wnt signaling and MED12 polymorphism could have profound effects on mental health and social behavior.

## RESULTS

### Med12 is required for *oxt* expression

**Fig. 1.**
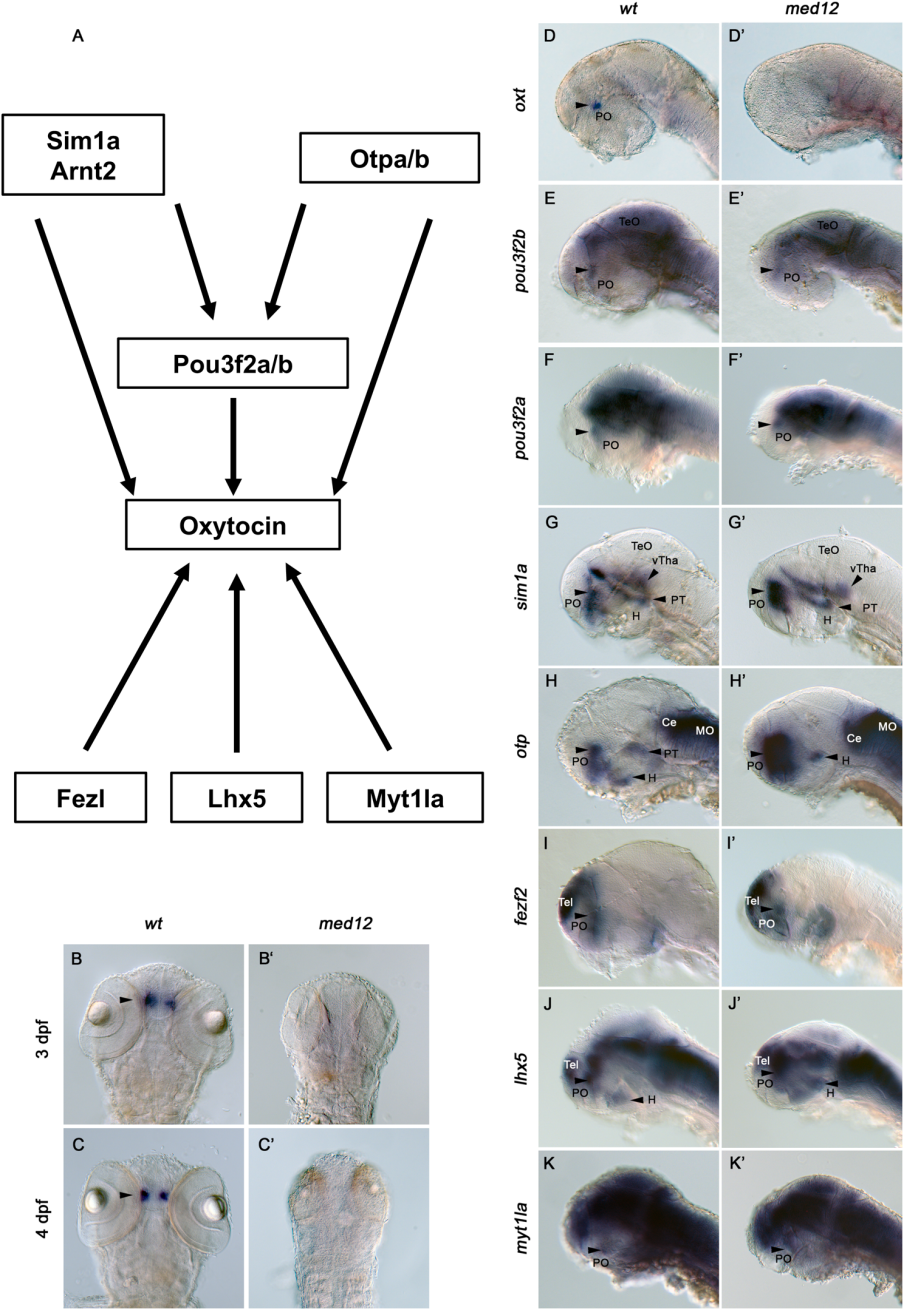
**Expression of *oxt* in the pre-optic area is undetectable in the *med12* mutant while expression of transcription factors required for *oxt* remain intact.** (A) A simplified diagram of the transcriptional network regulating oxytocin expression. Otpb, Sim1a, Pou3f2b, Fezl, and Lhx5 are all required for oxytocin expression. B-C′ are all dorsal views of *oxt* expression in the PO of WISH embryos magnified ×100. (B,B′) 72 hpf. (C,C′) 96 hpf. B,C are wild-type siblings. B′,C′ are *med12* mutants. D-K′ are all lateral views of 48 hpf WISH embryos magnified ×100. D,E,F,G,H,I,J,K are wild-type siblings. D′,E′,F′,G′,H′,I′,J′ are *med12* mutants. (D,D′) *oxt* expression in the PO. (E,E′) *pou3f2b* expression in the PO (F,F′) *pou3f2a* expression in the PO. (G,G′) *sim1a* expression in the vTha, PT, and PO. (H,H′) *otpb* expression in the H, PT, and PO. (I,I′) *fezl* expression in the PO and Tel. (J,J′) *lhx5* expression in the PO, Tel and H. (K,K′) *mytl1a* expression in the POA. PO, pre-optic area; TeO, optic tectum; vTha, ventral thalamus; PT, posterior tuberculum; H, hypothalamus; Ce, cerebellum; MO, medulla oblongata; Tel, telencephalon.

Loss-of-function *med12* mutations result in a wide range of brain defects in zebrafish embryos including deficits in hindbrain serotonergic neurons, locus coerulus (Lc) noradrenergic neurons, and dopaminergic neurons in the posterior tuberculum (PT) and PO (Wang et al., 2006; Guo et al., 1999). Moreover, deficits in PT dopaminergic neurons are due to *med12*-dependent elimination of *otp* expression in this brain region (Del Giacco et al., 2006). Because Otp is an upstream regulator of *oxt* expression, we examined whether *med12* mutations would disrupt *oxt* expression in the PO (Fig. 1A). We utilized the *med12^y82/y82^* mutant, which harbors a nucleotide change in a splice donor site that leads to splicing from a downstream site, which causes a frameshift and chain termination, presumably leading to production of a non-functional protein (Hong et al., 2005). In contrast to previous studies, which demonstrated reductions in the cell types examined, whole-mount *in-situ* hybridization (WISH) shows a loss of *oxt* expression in *med12* mutant embryos at 48 hpf (Fig. 1D,D′), 72 hpf (Fig. 1B,B′), and 96 hpf (Fig. 1C,C′). *med12* mutant embryos die around 4 to 5 dpf.

### Med12 is required for *oxt* expression independently of upstream transcriptional regulators

We and others have shown that the transcriptional regulators Pou3f2b, Sim1a and Otpb are required for *oxt* expression (Fig. 1A) (Eaton and Glasgow, 2006, 2007; Kasher et al., 2016; Blechman et al., 2007; Fernandes et al., 2013; Löhr et al., 2009). Thus, we asked whether loss of *oxt* expression in the *med12* mutant could be explained by loss of expression of *pou3f2b*, *sim1a* or *otpb*. WISH shows the presence of all three transcriptional regulators in the PO of *med12* mutant embryos. Expression of *pou3f2b* and *pou3f2a* is intact in the PO of the *med12* mutant (Fig. 1E,E′,F,F′). In addition, *sim1a* is expressed in a wild-type pattern in *med12* mutant embryos with staining seen in the ventral thalamus (vTha), PT, and PO, consistent with Borodovsky et al. (Fig. 1G,G′) (Borodovsky et al., 2009). Furthermore, *otpb* is expressed in the PO of *med12* mutants, even though expression is eliminated in the PT (Fig. 1H,H′). These results suggest that, in contrast to dopaminergic cell development in the PT, the effect of loss-of-function *med12* mutations on *oxt* expression is *otp-*independent.

Since *oxt* expression is undetectable in the *med12* mutant, alterations in pro-neuronal and brain patterning genes in the absence of Med12 are of interest. Wang et al. surveyed several brain patterning genes and expression of the pan-neuronal marker HuC, and no major deficits in brain patterning or global neuron number were found (Wang et al., 2006). In addition, we examined expression of forebrain markers *fezf2* and *lhx5*. Fezf2 is a forebrain-specific transcription factor involved in patterning and neurogenesis (Berberoglu et al., 2009). Additionally, Fezf2 is required for *oxt* expression in zebrafish (Blechman et al., 2007). Importantly, *fezf2* is expressed in the PO of the *med12* mutant, indicating that loss of *fezf2* is not responsible for loss of *oxt* expression in embryos lacking functional Med12 (Fig. 1I,I′). Lhx5 regulates forebrain development and is a transcriptional activator of Wnt antagonists (Peng and Westerfield, 2006). Lhx5 is also required for *oxt* expression (Machluf et al., 2011). WISH results suggest that expression of *lhx5* is intact in the PO of the *med12* mutant, indicating that alterations in *lhx5* are not responsible for loss of *oxt* in the *med12* mutant (Fig. 1J,J′).

Myt1la is a pro-neuronal transcription factor, the disruption of which has been associated with intellectual disability and obesity in human patients. Knock-down of *myt1la* in zebrafish results in loss of *oxt* expression (Blanchet et al., 2017). We find that *myt1la* is expressed in the PO of *med12* mutants, indicating that altered *mytl1a* expression is not responsible for the loss of *oxt* expression in the *med12* mutant (Fig. 1K,K′).

The observed lack of expression changes of transcription factors required for *oxt* expression in the PO of the *med12* mutants indicates that loss of *oxt* in the *med12* mutant is independent of known transcription factors controlling *oxt* expression. Although it is possible that post-48 hpf alterations in expression of the markers examined might contribute to loss of *oxt* expression in the *med12* mutant at later time points, it seems unlikely that any are solely responsible for the loss of *oxt* considering that *oxt* expression is already undetectable at 48 hpf in the *med12* mutant.

### Med12 affects multiple hypothalamic and pituitary cell types

**Fig. 2.**
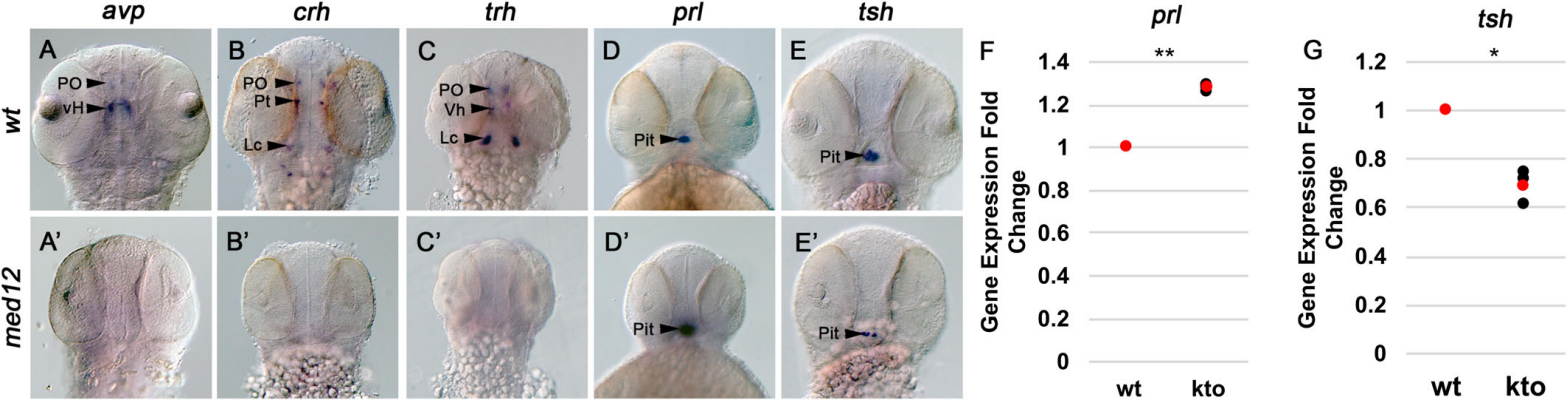
**Med12 is required for expression of *avp*,** ***crh* and *trh* and modulates expression of *prl* and *tsh*.** A-E′ are all dorsal views of WISH in 48 hpf embryos magnified ×100. (A,B,C,D,E) wild-type siblings. (A′,B′,C′,D′,E′) *med12* mutants. (A,A′) *avp* expression in the PO and vH. (B,B′) *crh* expression in the PO, PT and Lc. (C,C′) *trh* expression in the PO, vH and Lc. (D,D′) *prl* expression in the pituitary. (E,E′) *tsh* expression in the pituitary. (F,G) Expression of *prl* (F) and *tsh* (G) were measured in 48 hpf embryos using RT-qPCR. Gene expression values were normalized to an endogenous control, *beta actin 2* (*actb2*), and are expressed as fold changes relative to wild-type sibling controls. **P<0.01, *P<0.05, two-tailed paired Student's *t*-test. Dot plots show three biological replicates (black) and the mean (red). PO, pre-optic area; PT, posterior tuberculum; Lc, locus coerulus; vH, ventral hypothalamus; Pit, pituitary.

Because the *med12* mutation eliminated *oxt* expression completely while only causing reductions of serotonergic, noradrenergic and dopaminergic neurons at specific loci, we decided to survey additional cellular subtypes within the hypothalamic-pituitary axis. WISH shows that *arginine vasopressin* (*avp*) is undetectable in the *med12* mutant in both the PO and ventral hypothalamus (vH) (Fig. 2A,A′). Additionally, *corticotrophin-releasing hormone* (*crh*) expression is undetectable, with no expression in the PO, PT, or Lc of the *med12* mutant (Fig. 2B,B′), and *thyrotrophin-releasing hormone* (*trh*) expression is undetectable, with no expression in the PO, vH, or Lc (Fig. 2C,C′). However, not all cellular subtypes in the hypothalamic-pituitary axis are eliminated in the *med12* mutant; in the pituitary, *prolactin* (*prl*) expression is increased while *thyroid-stimulating hormone* (*tsh*) expression is reduced compared to wild type (Fig. 2D,D′,E,E′). Alterations in *tsh* and *prl* expression detected by WISH were confirmed using RT-qPCR (Fig. 2F,G). These data suggest that Med12 controls gene expression in multiple hypothalamic and pituitary cell types, presenting qualitatively different effects on several categories of cells. Serotonergic, noradrenergic and dopaminergic neurons are reduced, but only in specific brain nuclei, while *oxt*, *avp*, *crh* and *trh* expressing neurons are dependent on Med12 function in all areas of the brain. In the pituitary, the function of Med12 appears to be more complex and has both positive and negative effects on hormone gene expression. The mechanism for this effect is unknown, but one possibility is that Med12 affects key developmental signaling mechanisms such as the Wnt/β-catenin pathway.

In mice, expression of *Pit1* and markers of somatotropes (*Gh*), lactotropes (*Prl*) and thyrotropes (*Tshβ*) are dependent on Wnt signaling in the developing pituitary (Olsen et al., 2006). However, although the roles of Shh, Fgf, and Notch/Delta signaling in pituitary development have been well characterized in zebrafish, the role of Wnt signaling has not been well studied (Pogoda and Hammerschmidt, 2009). We find that in Wnt-inhibited embryos, the lactotrope marker *prl* becomes highly variable while the thyrotrope marker *tsh* is increased (Fig. S1A,A′,D,D′,F,H,J). Meanwhile, in the *apc* mutant, which has overactive Wnt signaling, expression of *prl* is increased while expression of *tsh* is decreased (Fig. S1B,B′,E,E′,G,I J). We conclude that Wnt signaling plays a critical role in cell type specification in the developing zebrafish pituitary, establishing a role for Wnt signaling in pituitary development across vertebrate species (Fig. S1J).

### Med12 is required for Wnt signaling during zebrafish development

Cell culture experiments indicate that MED12 physically interacts with β-catenin to activate transcription of Wnt-responsive genes (Kim et al., 2006). Additionally, *Med12* hypomorphic mouse embryos exhibit reduced expression of target genes of Wnt/β-catenin signaling (Rocha et al., 2010). Similarly, findings in fly and worm suggest that Med12 orthologs in these species are activators of Wnt signaling (Carrera et al., 2008; Yoda et al., 2005). In contrast, Lin et al. note that injection of a *med12* morpholino increases the proportion of embryos exhibiting a multiple tail bud phenotype induced by Wnt8 mRNA injection (Lin et al., 2007). This suggests an inhibitory role for Med12 in Wnt signaling and raises the question of whether Med12 inhibits, or activates Wnt signaling in zebrafish as it does in other model systems. However, although multiple studies have analyzed *med12* mutant zebrafish, none have specifically assessed the role of Med12 in Wnt signaling (Wang et al., 2006; Wu et al., 2014; Hong et al., 2005; Guo et al., 1999; Hong and Dawid, 2011). Here, we ascertain whether Med12 is required for Wnt signaling and transcription of Wnt target genes in zebrafish.

**Fig. 3.**
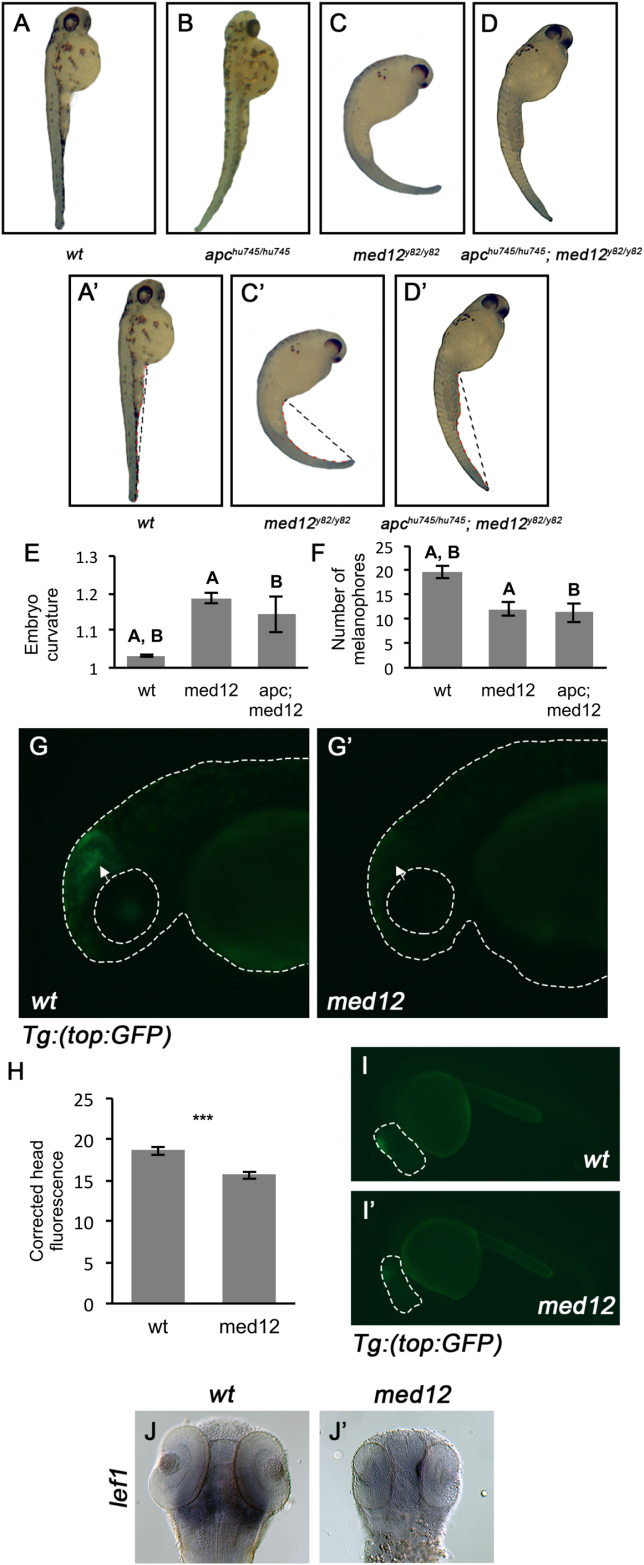
**Med12 is epistatic to Apc and is required for Wnt-dependent gene expression.** A-D are all lateral views of 48 hpf embryos. (A,A′) wild-type embryos. (B) *apc^hu745/hu745^* mutant. (C,C′) *med12^y82/y82^* mutants. (D,D′) *apc ^hu745/hu745^*; *med12^y82/y82^* double mutant. In A′,C′ and D′, the black dotted line represents the distance from the yolk to the tail tip and the red dotted line represents the tail length. (E) Tail curvature of *apc ^hu745/hu745^*; *med12^y82/y82^* double mutants was compared to that of wild-type and *med12^y82/y82^* mutant embryos. Tail curvature is the tail length divided by the distance from yolk to tail tip. wild type: *n*=20, *med12^y82/y82^*: *n*=24, *apc ^hu745/hu745^*; *med12^y82/y82^*: *n*=8. A, *P*<0.001; B, *P*<0.05, two-tailed unpaired Student's *t*-test. Error bars show s.e.m. (F) The number of melanophores on the lateral surface of the yolk of *apc ^hu745/hu745^*; *med12^y82/y82^* double mutants was compared to that of wild-type and *med12^y82/y82^* mutant embryos. wild type: *n*=15, *med12^y82/y82^*: *n*=27, *apc ^hu745/hu745^*; *med12^y82/y82^*: *n*=9. A,B *P*<0.001, two-tailed unpaired Student's *t*-test. Error bars show s.e.m. (G,G′) Representative images of *GFP* expression visualized at ×100 magnification in 24 hpf wild-type siblings and *med12* mutants that are heterozygous for the *TOP:GFP* transgene. Arrows indicate area of reduced GFP expression. (H) Corrected head fluorescence was compared between wild-type and *med12^y82/y82^* mutant embryos. Corrected head fluorescence is head fluorescence (Integrated Density) divided by the area of the head. wild type: *n*=8, *med12^y82/y82^*: *n*=9. *P*<0.001, two-tailed unpaired Student's *t*-test. Error bars show s.e.m. (I,I′) Representative full embryo views of *GFP* expression magnified ×50 in 24 hpf wild-type siblings and *med12* mutants that are heterozygous for the *TOP:GFP* transgene. Dashed line indicates an example of the area (the head) that was circled for fluorescence and area measurements in H. J,J′ are dorsal views of WISH for *lef1* in 48 hpf embryos magnified ×100. (J) wild-type sibling. (J′) *med12* mutant.

We in-crossed *apc^hu745/+^*; *med12^y82/+^* embryos and found that embryo phenotypes did not conform to expectations for a dihybrid cross. The *apc^hu745/hu745^* and *med12^y82/y82^* embryos have easily identifiable phenotypes at 48 hpf; while the *apc^hu745/hu745^* mutant exhibits a dorsal tail curve, the *med12^y82/y82^* mutant can be identified by a pronounced lateral tail curve (Fig. 3A,B,C). The proportion of *med12* phenotypes from the dihybrid cross was higher than expected (χ^2^=54.770, *P*<0.0001). We reasoned that if Med12 is downstream of and epistatic to Apc, the *apc^hu745/hu745^*; *med12^y82/y82^* embryos would resemble the *med12^y82/y82^* mutants phenotypically, thus explaining the observed phenotype ratios. We characterized the morphology of wild-type, *med12^y82/y82^* and *apc^hu745/hu745^*; *med12^y82/y82^* embryos at 48 hpf by measuring their tail curvature as calculated by dividing the total length of the tail from the bottom of the yolk to the tail tip, by the distance from the bottom of the yolk to the tail tip (Fig. 3A′,C′,D′,E). The *apc^hu745/hu745^*; *med12^y82/y82^* embryos exhibited a lateral tail curve and not a dorsal tail curve (Fig. 3D). The tail curve measurements of *apc^hu745/hu745^*; *med12^y82/y82^* embryos overwhelmingly resembled *med12^y82/y82^* embryos as compared with *apc^hu745/hu745^* mutants and wild-type embryos (Fig. 3E). It has been previously reported that *med12^y82/y82^* mutants exhibit a reduction in melanophores at 48 hpf, likely due to a failure in migration of neural crest cells, which are melanocyte precursors (Hong et al., 2005). We counted the number of melanophores on the lateral side of wild-type, *med12^y82/y82^* mutant and *apc^hu745/hu745^*; *med12^y82/y82^* double mutants. We observed a statistically significant reduction in melanophore number in both *med12^y82/y82^* mutants and *apc^hu745/hu745^*; *med12^y82/y82^* double mutants as compared with wild type (Fig. 3F). These data suggest that Med12 is epistatic to Apc, as *apc^hu745/hu745^*; *med12^y82/y82^* mutants resemble *med12^y82/y82^* mutants phenotypically.

To provide further evidence that Med12 is required for Wnt signaling in zebrafish, we utilized the *Tg(TOP:GFP)* transgenic reporter line, in which *GFP* expression is driven by a Wnt-responsive promoter (Dorsky et al., 2002). At 24 hpf, when the *TOP:GFP* transgene is normally highly expressed, *GFP* expression was reduced in *med12* mutants heterozygous for the *TOP:GFP* transgene as compared with wild type, indicating that Med12 is required for Wnt signaling (Fig. 3G,G′,H,I,I′). Additionally, expression of the Wnt-responsive gene *lef1* is reduced in the *med12* mutant as compared with wild type (Fig. 3J,J′) (Kawakami et al., 2006).

### Med12 is required for oxytocin expression independently of Wnt signaling

**Fig. 4.**
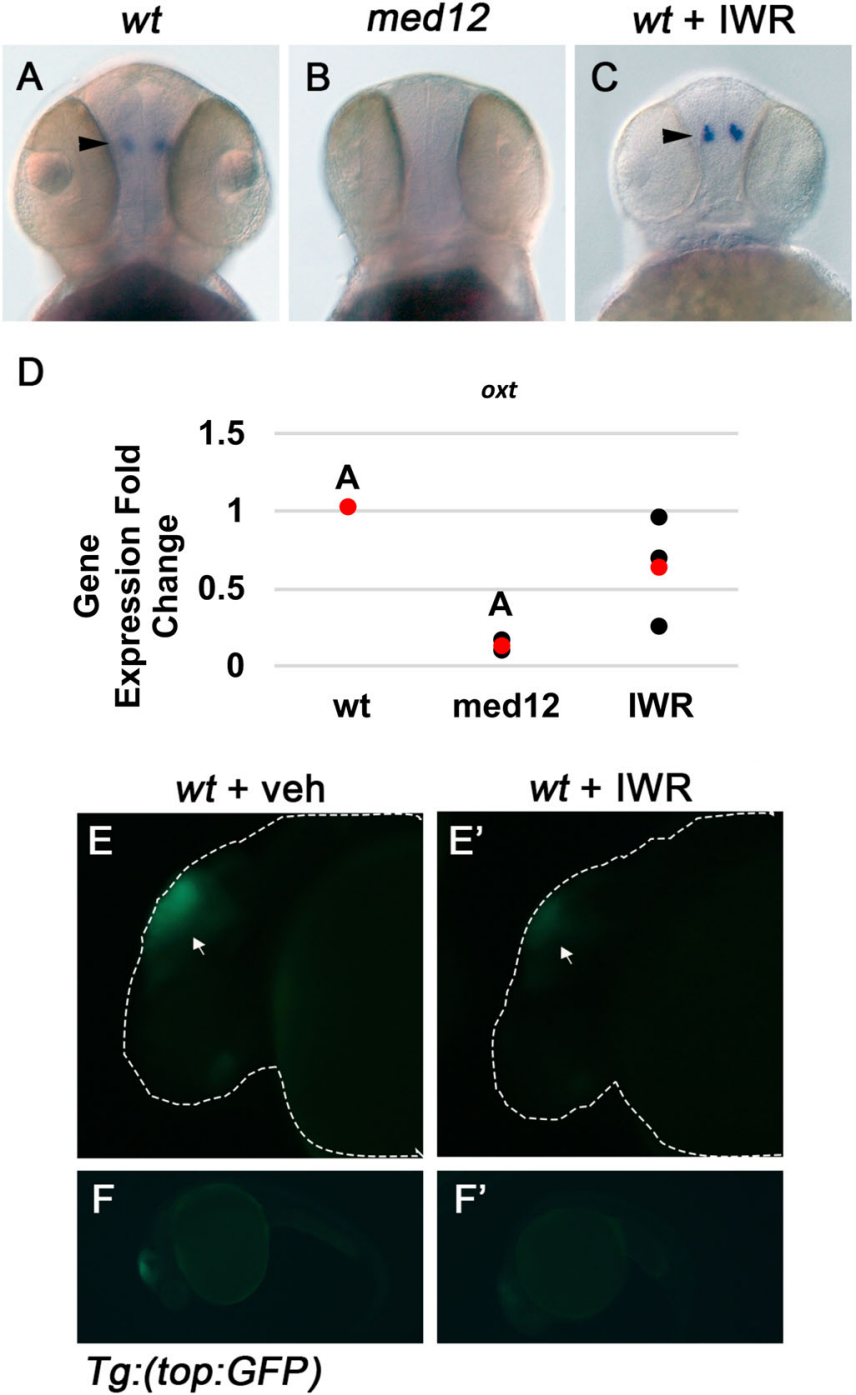
**Expression of *oxt* is undetectable in the *med12* mutant but is unaffected by treatment with Wnt inhibitors.** A-C are all dorsal views of WISH for *oxt* in 48 hpf embryos magnified ×100 in which arrowheads indicate *oxt* expression. Embryos treated with IWR and *med12* mutants were compared to sibling controls. (A) wild-type embryo. (B) *med12* mutant embryo. (C) IWR-treated embryo. (D) *oxt* expression was measured using RT-qPCR in 48 hpf embryos. Gene expression values were normalized to an endogenous control, *beta actin 2* (*actb2*), and are expressed as fold changes relative to wild-type sibling, or vehicle-treated wild-type sibling controls. A: *P*<0.01, two-tailed paired Student's *t*-test. Dot plot shows three biological replicates (black) and the mean (red). (E,E′,F,F′) Representative images of *GFP* expression was visualized in 24 hpf vehicle and IWR-treated embryos that are heterozygous for the *TOP:GFP* transgene. (E,E′) Magnified ×100. Arrows indicate area of reduced *GFP* expression. (F,F′) Representative full embryo views magnified ×50.

Although Med12 is required for proper expression of numerous cell-type specific markers, we focused on *oxt* expression in particular because altered oxytocinergic systems are linked to behavioral and appetite-related disorders. Because Med12 plays a role in Wnt signaling during zebrafish development, we investigated potential roles of Wnt signaling in oxytocin expression. To inhibit Wnt signaling, we used IWR, which inhibits Tankyrase enzymes that regulate turnover of Axin proteins. In the absence of Tankyrase activity, Axin accumulates and promotes destruction of β-catenin, thereby reducing transcription of Wnt-responsive genes. IWR was previously shown to inhibit tailfin regeneration and reduce expression of a Wnt-responsive 7x TCF-*siam*:EGFP transgene in zebrafish embryos (Dodge et al., 2012). We achieved similar results when treating *Tg (TOP:GFP)* transgenic reporter fish with IWR (Fig. 4E,E′,F,F′). Treatment with IWR also caused lateral tail curvature, a phenotype characteristic of *med12* mutant embryos (data not shown). Although *oxt* expression is undetectable in the PO of the *med12* mutant at 48 hpf, *oxt* expression is unaffected in embryos treated with IWR, suggesting that Med12 is required for oxytocin expression independently of Wnt signaling (Fig. 4A-C). RT-qPCR was used to confirm that *oxt* expression is reduced in *med12* mutants, but not in IWR treated embryos (Fig. 4D).

### Overactive Wnt Signaling suppresses oxytocin expression

**Fig. 5.**
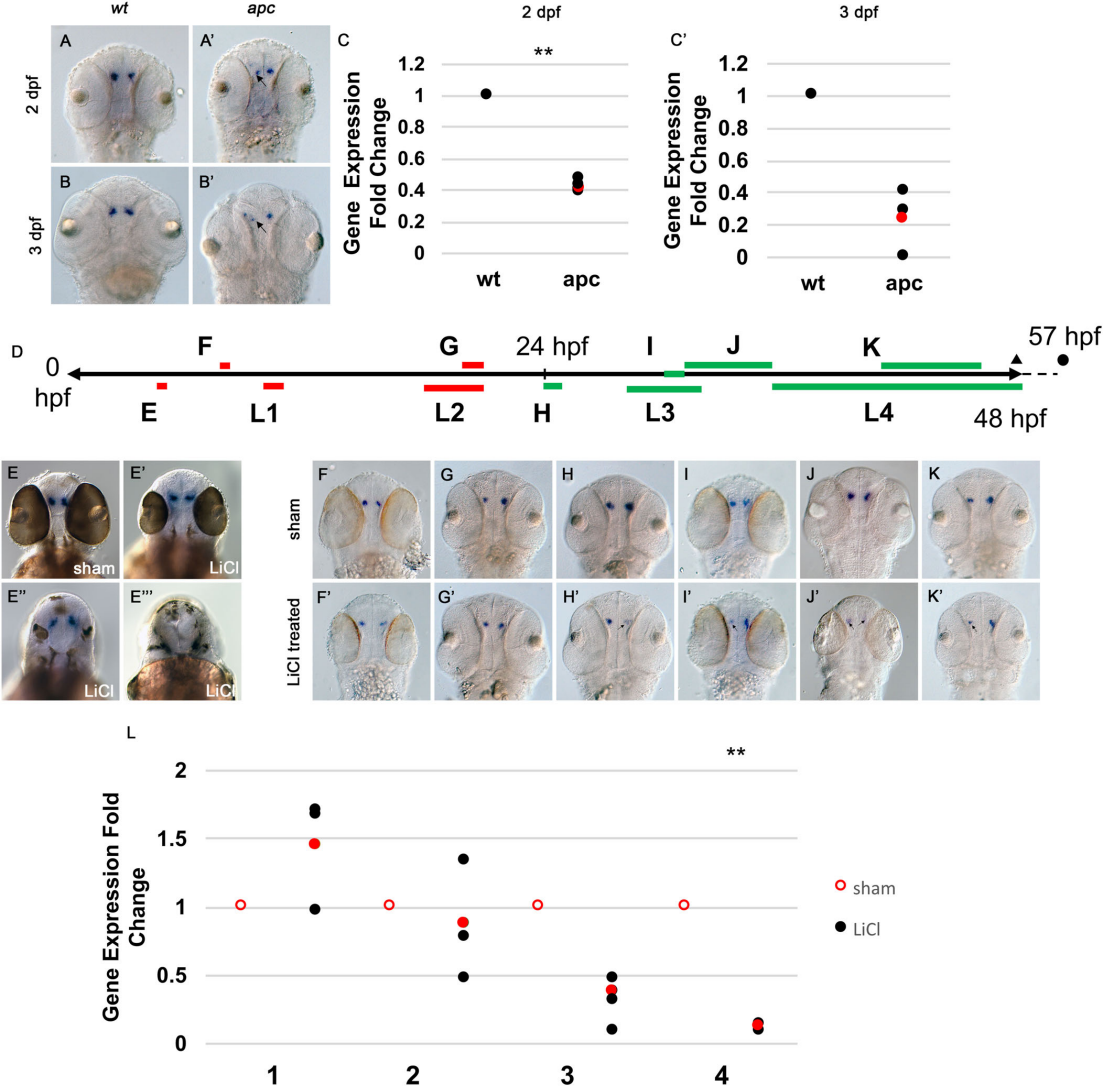
**Expression of *oxt* is reduced in the *apc* mutant and LiCl-treated embryos starting at 24 hpf.** A-B′ are all dorsal views of WISH for *oxt* magnified ×100. (A,A′) 48 hpf embryos. (B,B′) 72 hpf embryos. (A,B) wild-type siblings. (A′,B′) *apc* mutants in which arrows indicate areas of reduced *oxt* expression. (C,C′) *oxt* expression was measured at 48 and 72 hpf in wild-type and *apc* mutant embryos using RT-qPCR. Gene expression values were normalized to an endogenous control, *beta actin 2* (*actb2*), and are expressed as fold changes relative to wild-type sibling controls. **P<0.01, two-tailed paired Student's *t*-test. Dot plots show three biological replicates (black) and the mean (red). (D) Timeline schematic showing LiCl treatment time and duration. Lettering in the schematic corresponds to labeling in the figure. Triangle represents fixation point for treatments E,F,G,H,I,K and L. Circle represents fixation point for treatment J. Red, no effect on *oxt* expression. Green, leads to reduced *oxt* expression. E,E′,E″,E′″ are all dorsal views of WISH for *oxt* magnified ×100. E-K′ are all dorsal views of WISH for *oxt* in wild-type embryos magnified ×100. E,F,G,H,I,J,K underwent sham treatment in fish water. E′,E″,E″′,F′,G′,H′,I′,J′,K′ were treated with LiCl. Arrows in F′,G′,H′,I′,J′,K′ indicate areas of reduced *oxt* expression. (E-K′) 48 hpf. (J,J′) 57 hpf. (E-E′″) treated at 5 hpf for 20 min. (F,F′) treated at 8 hpf for 30 min. (G,G′) treated at 20 hpf for 1 h. (H,H′) treated at 24 hpf for 1 h. (I,I′) treated at 30 hpf for 1 h. (J,J′) treated at 31 hpf for 4.5 h. (K,K′) treated at 41 hpf for 5 h (L) *oxt* expression was measured in LiCl and sham treated embryos at 48 hpf using RT-qPCR. 1: treated at 10 hpf for 1 h; 2: treated at 18 hpf for 3 h; 3: treated at 28 hpf for 3.75 h. 4: treated at 36 hpf for 12 h. Gene expression values were normalized to an endogenous control, *beta actin 2* (*actb2*), and are expressed as fold changes relative to sham-treated wild-type sibling controls. **P<0.01, two-tailed paired Student's *t*-test. Dot plot shows three biological replicates (black) and the mean (red).

Surprisingly, although Wnt inhibition had no effect on *oxt* expression, *oxt* expression is reduced in *apc* mutants, which have overactive Wnt signaling. Reduced *oxt* expression in the *apc* mutants as detected by WISH was evident at 48 hpf (Fig. 5A,A′). This reduction appeared more pronounced at 72 hpf (Fig. 5B,B′). Reductions in *oxt* expression in the *apc* mutant at 48 hpf and 72 hpf were confirmed by RT-qPCR (Fig. 5C,C′). Despite expression pattern changes in other nuclei, *sim1a* and *otpb* are expressed in the PO of the *apc* mutant at 48 hpf, indicating that reduction of *oxt* expression is independent of these transcription factors (Fig. S2B,B′,C,C′). Although *pou3f2b* is expressed in the PO of the *apc* mutant, expression is reduced in the PO and more diffuse throughout the embryo as compared with wild type, suggesting that a reduction in *pou3f2b* might underlie the reduction in *oxt* observed in the *apc* mutant (Fig. S2A,A′).

Although it is not known when oxytocin cells become specified, it might be around 14 hpf when nearby dopaminergic cells in the PO are specified (Russek-Blum et al., 2008). The start of *oxt* expression, which is the defining step in the differentiation of oxytocin cells, likely occurs just prior to 36 hpf, when a low level of *oxt* is detectable by WISH (Unger and Glasgow, 2003). We asked which stage of oxytocin cell development is disrupted by overactive Wnt signaling.

Embryos were treated at various time points throughout development with LiCl, a chemical activator of Wnt signaling that works by inhibiting GSK3β and stabilizing β-catenin, which activates transcription of Wnt target genes (Robertson et al., 2014) (Fig. 5A). LiCl treatment differs in lethality depending on developmental stage, suggesting different sensitivities to LiCl (Table S1). Thus, treatment duration was adjusted depending on the stage of the embryo. We sought to treat embryos prior to 14 hpf to assess whether overactive Wnt signaling disrupts *oxt* expression early in *oxt* cell development. Only 55% of embryos survived LiCl treatment at 5 hpf for 20 min, while 0% survived treatment at 6 hpf for 12 h and 9 hpf for 2 h. However, 83.3% of embryos survived LiCl treatment at 8 hpf for 30 min and 100% of embryos survived treatment at 10 hpf for 1 h (Table S1). Therefore, we performed WISH for *oxt* on embryos treated with LiCl at 5 hpf for 20 min or 8 hpf for 30 min, and RT-qPCR on embryos treated at 10 hpf for 1 h. LiCl treatment at 5 hpf for 20 min causes a variable posteriorization phenotype; that is, some embryos are posteriorized while others are not (Fig. 5E,E′,E″,E′″). In this experiment, non-posteriorized embryos always had unaffected *oxt* expression as detected by WISH (Fig. 5E′). Even among LiCl-treated embryos that lost their eyes, *oxt* expression was often still present (Fig. 5E″). This was not the case in extreme examples, presumably because the PO is no longer present in these embryos (Fig. 5E′″). Additionally, WISH shows that 30 min LiCl treatment at 8 hpf has no effect on *oxt* expression (Fig. 5F,F′). RT-qPCR reveals no significant differences in *oxt* expression for embryos treated with LiCl at 10 hpf for 1 h and controls (Fig. 5L).

Since early treatment with LiCl did not result in a reduction in *oxt* expression, we sought to identify the stage at which LiCl treatment would begin to affect *oxt*. To that end, embryos were treated with LiCl for 1 h at 20 hpf, 24 hpf and 30 hpf. Since embryos appear to be more sensitive to LiCl at earlier developmental stages, we reasoned that the 20 hpf treatment would either be stronger or the same as the 24 hpf treatment and the 30 hpf treatment. However, *oxt* appeared to be reduced after 1 h LiCl treatment at 24 hpf and 30 hpf, but not after 1 h LiCl treatment at 20 hpf, suggesting that LiCl begins to affect *oxt* around 24 hpf (Fig. 5G,G′,H,H′,I,I′). To test whether a stronger pre-24 hpf LiCl treatment would affect *oxt* expression, we treated embryos at 18 hpf for 3 h, then performed RT-qPCR. No significant difference in *oxt* expression was found between embryos treated with LiCl at 18 hpf for 3 h and controls (Fig. 5L). To test whether post-24 h LiCl treatment reduces *oxt* expression at other treatment stages and durations, we treated 28 hpf embryos for 3.75 h, 31 hpf embryos for 4.5 h, 36 hpf embryos for 12 h, and 41 hpf embryos for 5 h. Using WISH, reductions in *oxt* expression were detected after LiCl treatment at 31 hpf for 4.5 h and 41 hpf for 5 h (Fig. 5J,J′,K,K′). RT-qPCR revealed decreases in *oxt* expression for embryos treated with LiCl at 28 hpf for 3.75 h and at 36 hpf for 12 h (Fig. 5L). Together, these results suggest that overactive Wnt signaling suppresses *oxt* expression starting around 24 hpf. Since *oxt* is detectable by WISH starting around 36 hpf, overactive Wnt signaling might interfere with the final steps of *oxt* cell differentiation. Thus, Wnt signaling must be suppressed at the 24 hpf stage to allow for proper *oxt* cell development, of which expression of *oxt* is the defining step.

## DISCUSSION

### Differing mechanisms for Med12 across cellular subtypes

Previous work by Wang et al. demonstrates that forebrain patterning in the *med12* mutant is grossly normal, with expression of the pan-neuronal marker HuC largely unperturbed (Wang et al., 2006). Similarly, we find that expression of hypothalamic markers *pou3f2*, *sim1*, *otp*, *fezf2*, and *lhx5* are expressed normally in the preoptic area of *med12* mutant embryos. However, cell-type specific gene expression is severely impacted. Our finding that expression of *oxt*, *avp*, *crh*, and *trh* are undetectable in the *med12* mutant contrasts with data showing that pituitary markers are only upregulated or downregulated, and with studies of *med12* mutants which indicate that dopaminergic and serotonergic cells are reduced, but not eliminated (Wang et al., 2006; Guo et al., 1999). These differing results across cell types may indicate divergent mechanisms for Med12.

Wang et al. suggest that reductions in forebrain and hindbrain dopaminergic, and hindbrain serotonergic neuron subsets in the *med12* mutant are due to loss of expression of the neural determination genes *ascl1a* and *lhx1*. Expression of *ascl1a* and *lhx1* are decreased in the *med12* mutant and injection of *med12* RNA led to increased expression of both transcription factors (Wang et al., 2006). It seems unlikely, however, that loss of *ascl1a* or *lhx1* is responsible for the loss of *oxt*, *avp*, *crh*, and *trh* that we observed in the *med12* mutant, because the *ascl1a* and *lhx1* expression patterns do not appear to overlap with all of the relevant expression domains in the PO, vH, PT and Lc (Thisse and Thisse, 2005). While reductions in dopaminergic and serotonergic neurons may be due to subtle defects in brain patterning or cell fate determination in the *med12* mutant, complete loss of *oxt*, *avp*, *crh* and *trh* expression suggests a more direct role for Med12 and leaves open the possibility that Med12 is required for transcription of these genes. Future work could determine whether loss of Med12 in *oxt* cells alone is enough to eliminate *oxt* gene expression, thus elucidating whether the role of Med12 in *oxt* expression is cell autonomous.

Previous work suggests that the reduction in *tyrosine hydroxylase* (*th*) expression in the PT of the *med12* mutant is caused by loss of *otp* expression in the PT (Del Giacco et al., 2006). However, considering that *otp* is expressed in the PO of the *med12* mutant, it does not appear that loss of *otp* is responsible for its loss of *oxt* expression. Although *crh* is expressed in the PT, it is unlikely that loss of *otp* underlies loss of *crh* in the *med12* mutant because in the *med12* mutant, *crh* expression is lost not only in the PT but in all nuclei (Fig. 2B′). Altogether, loss of *otp* does not appear to be responsible for the gene expression alterations that we observe in the *med12* mutant. Combined, our data highlight the complexity of the role of Med12 in vertebrate development and cell type-specific gene expression.

### An *in-vivo* role for Med12 as an activator of Wnt signaling

The role of Med12 in transcriptional regulation is an active area of investigation in several contexts. Work in yeast suggests that the CDK8 complex, of which MED12 is a part, sterically blocks Mediator interaction with Pol II, thereby acting as a transcriptional repressor (Elmlund et al., 2006). However, a study of acute myeloid leukemia cells found that silencing of MED12 is associated with transcriptional repression (Bhagwat et al., 2016). In zebrafish the only published study on the role of Med12 in Wnt signaling suggests that Med12 is a repressor of Wnt signaling (Lin et al., 2007), although wnt1 and wnt8b genes have been shown to be variously down-regulated in the hindbrain of Med12 mutants (Hong and Dawid, 2011). Here we show that in zebrafish Med12 is epistatic to Apc, a component of the Wnt pathway, and that expression of a Wnt reporter is reduced in the *med12* mutant, supporting a role for Med12 as an activator of Wnt signaling in zebrafish. Although our data suggest that reductions in Wnt signaling are not responsible for the loss of *oxt* expression observed in the *med12* mutant, dysregulated Wnt signaling likely contributes to other facets of the *med12* mutant phenotype.

Although pituitary marker expression changes in Wnt-inhibited embryos are not consistent with those in the *med12* mutant, which is Wnt-inhibited (Fig. S1J), this might be because IWR treatment is expected to affect all Wnt signaling, while Med12 may only be involved in Wnt signaling though Tcf/Lef1. In mouse, β-catenin was found to also interact with the homeodomain factor Prop1, rather than only Tcf/Lefs, to activate transcription of the pituitary lineage-determining factor *Pit1* (Olson et al., 2006). Pit1, in turn, is required for lactotropes, thyrotropes, and somatotropes in zebrafish (Herzog et al., 2004). Our results suggest that altered Wnt signaling may reduce specification of some pituitary cell types, thus allowing other cell types to inappropriately expand. Moreover, these data indicate that the role of Wnt signaling in pituitary development is an area ripe for study in the zebrafish model.

### Identification of a Wnt-repressive center necessary for *oxt* expression

We found that *oxt* expression is not reduced in Wnt-inhibited embryos. In fact, *oxt* expression appears to be reduced in *apc* mutants, which have overactive Wnt signaling. Interestingly, in *histone deacetylase 1* (*hdac1*) zebrafish mutants, which also have overactive Wnt signaling, *oxt* expression is eliminated (Nambiar and Henion, 2004; E.G., unpublished data). Together, these data suggest that Wnt signaling needs to be suppressed for *oxt* cells to develop properly. Our chemical Wnt activation experiments suggest that Wnt repression is not required until around 24 hpf, suggesting a role in *oxt* cell differentiation, rather than specification.

Wnt signaling is known to play a role in early induction and patterning of the hypothalamus. Wnt ligands secreted from the posterior neuroectoderm promote hindbrain fate, and are opposed by Wnt antagonists secreted from the anterior neuroectoderm, creating an anterior-posterior gradient of Wnt signaling (Kudoh et al., 2002; Heisenberg et al., 2001; Pearson and Placzek, 2013). Kapsimali et al. identify a role for Wnt inhibition by showing that it is required to promote hypothalamic identity at the expense of floorplate identity as early as the 1-2 somite stage (Kapsimali et al., 2004). However, our data showing that overactive Wnt signaling suppresses *oxt* expression starting at 24 hpf suggests an effect on the differentiation of progenitor cells into specific neural subtypes, rather than an early effect on acquisition of hypothalamic identity. Our data suggest that overactive Wnt signaling promotes a progenitor-like state, and prevents *oxt* cells from fully differentiating. One way that overactive Wnt signaling might affect *oxt* cell development is by altering expression of transcription factors that promote *oxt* expression. In fact, we found that expression of *pou3f2b*, which is required for *oxt* expression, is reduced in the PO of the *apc* mutant, suggesting that overactive Wnt signaling suppresses *pou3f2b* expression (Fig. S2A,A′). Moreover, *pou3f2b*, although expressed regionally in the diencephalon starting around the 7-somite stage, becomes concentrated in the PO between 24 and 36 hpf, which is the same developmental stage in which *oxt* cells become sensitive to overactive Wnt signaling (Spaniol et al., 1996; Kasher et al., 2016). This suggests that reduced *pou3f2b* in the *apc* mutant could underlie its reduced *oxt* expression phenotype.

Since our data suggest that overactive Wnt signaling interferes with *oxt* expression, further investigation should explore whether activation of Wnt antagonists in the PO are also required for *oxt*. Interestingly, *lhx5*, which was shown to inhibit Wnt signaling up to the 16 somite stage and to rescue forebrain deficiencies caused by ectopic Wnt signaling, is expressed in the PO at 48 hpf (Peng and Westerfield, 2006; Manoli and Driever, 2014). Moreover, Lhx5 is required for *oxt* signaling in zebrafish (Machluf et al., 2011). However, ascertaining whether the role of *lhx5* in *oxt* cell development is dependent on Wnt inhibition awaits further study.

This study highlights the diverse roles of the Wnt signaling pathway and the transcriptional co-activator Med12 in regulating *oxt* expression in a vertebrate organism. Together our findings help to elucidate the role of signaling pathways and transcriptional regulators in controlling expression of *oxt*, the dysregulation of which can contribute to behavioral and appetite-related disorders.

## MATERIALS AND METHODS

### Animals

Zebrafish (*Danio rerio*, mixed background) were raised, maintained and crossed as previously described (Westerfield, 1995). Embryos developed at 28°C and were staged according to both hpf and morphological characteristics (Kimmel et al., 1995). All procedures were performed in accordance with NIH guidelines on the care and use of animals and were approved by the Georgetown University Institutional Animal Care and Use Committee, Protocol #100517.

### Genotyping

The *apc^hu745^* and *med12^y82^* mutations were genotyped either by heterozygote in-crosses or by using a custom-designed TaqMan SNP genotyping assay (Applied Biosystems, Foster, USA). The following sequences were submitted for primer design:

apc^hu745^:GCCAGTAACTACCCAACTTTACCTATATCAGAAAAACAATCCACTAATAATGTTGCAGCTGAC[C/T]AACGCACATCTGAGAGCCAATCATCAGTCCATTATGTTCGCGCCAAGCCTCCACGACATCATTTAG

*kto^y82^*: TCAGGATACTGGTATGTCCGAGTCGATGGAGATAGATCACAACTCAAGCGCTAACTTTGATGAG[G/A]TTGGTAAGAGGGTATTTTGTGTTAGTGATGTTTAACATAAGGCTGTATATAATCG

Genotyping reactions containing TaqMan Genotyping Master Mix (Applied Biosystems) were prepared according to the manufacturer's protocol and analyzed using the Applied Biosystems 7900HT Fast Real-Time PCR System.

### Drug treatments

For all drug treatments, embryos were collected from pair-wise or group mating, debris and unfertilized eggs were removed, and embryos were incubated in a 96-well plate containing 100 µl of drug solution. IWR solutions were prepared by dissolving 5 mg of IWR (Sigma-Aldrich) in 1.22 ml DMSO (Sigma-Aldrich) and then diluting this solution in fish water (0.3 g/l Instant Ocean Sea Salt, Pentair, Apopka, USA) with or without 30 mg/l phenylthiourea (Sigma-Aldrich). Embryos were incubated at a final concentration of 10 µM of IWR or vehicle solution starting at 4 hpf until fixation or RNA isolation. LiCl solutions were prepared by diluting LiCl (Sigma-Aldrich) in fish water with or without 30 mg/l phenylthiourea (Sigma-Aldrich). Embryos were incubated in a 0.3 M solution of LiCl or fish water at various time points.

### Whole-mount *in-situ* hybridization (WISH)

This study assessed expression of *oxt*, *sim1*, *pou3f2*, *otp*, *avp*, *trh*, *crh*, *tsh*, *prl*, *fezf2*, *lhx5*, *myt1l*, and *lef1* using WISH. WISH procedures were performed as previously described (Eaton and Glasgow, 2006). WISH was repeated at least two times using eight to 12 embryos per group. Control and experimental groups were processed in the same reaction, marking the sibling controls by cutting their tails. Synthesis of *oxt*, *sim1*, *pou3f2*, *otp*, *avp*, *lhx5*, *myt1la*, and *lef1* riboprobes have been described previously (Unger and Glasgow, 2003; Eaton and Glasgow, 2006, 2007; Kasher et al., 2016; Toyama et al., 1995; Eaton et al., 2008; Blanchet et al., 2017; Dorsky et al., 1999). The *trh* and *crh* probes were generated by PCR using F1 and T7 primers; forward and reverse primers were designed and purchased (Sigma-Aldrich) with the below sequences:

TRH_F1: 5′ CGAAGCGACAGTTCAAAGCC

TRH_R1-T7: 5′ TAATACGACTCACTATAGGGGCAAAAGTGCGAGATCCGTG

CRH_F1: 5′ TACGCACAGATTCTCCTCGC

CRH_R1-T7: 5′ TAATACGACTCACTATAGGGCTGATGGGTTCGCTTGTGGTT

Embryo cDNA was synthesized by reverse transcription from 2 dpf embryo RNA as per protocol for oligo dT20 primed Superscript kit (Invitrogen). PCR reactions [1 µl cDNA; 0.4 µM TRH-F1 or CRH_F1; 0.4 µM TRH-R1-T7 OR CRH-R1-T7; 1× PCR mix (Thermo Fisher Scientific)] were amplified using the following parameters for TRH: 94, 2 min; 15 cycles of 94, 30 s; 62, 10 s; 72, 1 min; 72, 10 min; 4, hold and for CRH: 94, 2 min; 30 cycles of 94, 30 s; 60, 30 s; 68, 30 s; 68, 10 min; 4, hold. Amplified product was purified using QIAquick PCR purification kit as per the manufacturer's instructions, followed by ethanol precipitation and resuspension in dH_2_O for subsequent DIG-labeled *in vitro* transcription (Roche, Basel, Switzerland). The *tsh* and *prl* riboprobes were synthesized from cDNA that has been described previously (Herzog et al., 2003). *tsh* was linearized with HindIII and *prl* with Kpn1. Both were ethanol precipitated and re-suspended in dH_2_O for DIG labeled *in vitro* transcription. The *fezf2* clone from Musahiko Hibii, Riken Center, Kobe, Japan, was subcloned into Bluesript II SK- (Stratagene, La Jolla, USA). Antisense riboprobe was made by linearizing the plasmid with SalI and performing DIG-labeled *in vitro* transcription with T3 RNA polymerase.

### RNA isolation and real time quantitative RT-qPCR

RNA was isolated from groups of 50 embryos using TRIzol Reagent (Invitrogen) and the Direct-zol RNA MiniPrep kit (Zymo Research, Irvine, USA). 1 µg RNA was then reverse transcribed using SensiFAST cDNA Synthesis kit (Bioline, Boston, USA). Reactions containing Fast SYBR Green master mix (Applied Biosystems), cDNA, and primers were amplified using an Applied Biosystems StepOnePlus PCR machine according to the manufacturer's protocol. The following primers were used:

TSHBA_F1 5′- CTGTCAACACCACCATCTGC

TSHBA_R1 5′- GTGCATCCCCTCTGAACAAT

PRL_F1 5′- ACATCAACAGTCTGGGAGCA

PRL_R1 5′- AAGACGAGCCCATCTTGTGT

OXT_F1 5′- GGTGTCAGCCTTGGTGAATAAT

OXT_R1 5′- GTTTGAGATGTAGCAGGCCG

Relative Quantitation was analyzed in RQ Manager 2.3 software using the Comparative C_T_ method.

### Microscopy

To obtain images of WISH staining, each embryo was mounted under a coverslip in a 1.5% agarose, 70% glycerol solution. For lateral views, eyes were removed from embryos prior to mounting. Images were acquired using a Zeiss Axioplan2 microscope fitted with an AxioCam camera using AxioVision software. To obtain images of transgenic embryos, live embryos were arranged on a slide in a 3% solution of methyl cellulose, and images were acquired using an Olympus IX-71 inverted microscope. Fluorescence and area measurements were made using Fiji (Schindelin et al., 2012).

### Statistics

Two-tailed unpaired Student's *t*-tests were used to analyze tail curvature and melanophore counts in wild-type and mutant embryos. A Chi-square Goodness-of-Fit test was performed to analyze the result of the *apc^hu745/+^*, *med12^y82/+^* in-cross. For RT-qPCR, ΔCt values were used for statistical analysis, and analyzed by two-tailed paired *t*-tests.

## Supplementary Material

Supplementary information
