# Supplementary material for: Separate roles for Med12 and Wnt signaling in regulation of oxytocin expression
Source: Biol Open. 2018 Mar 12;7(3):bio031229. doi: 10.1242/bio.031229 (PMC5898263; doi:10.1242/bio.031229)
Supplement: Supplementary information [file biolopen-7-031229-s1.pdf]

Biology Open (2018): doi:10.1242/bio.031229: Supplementary information

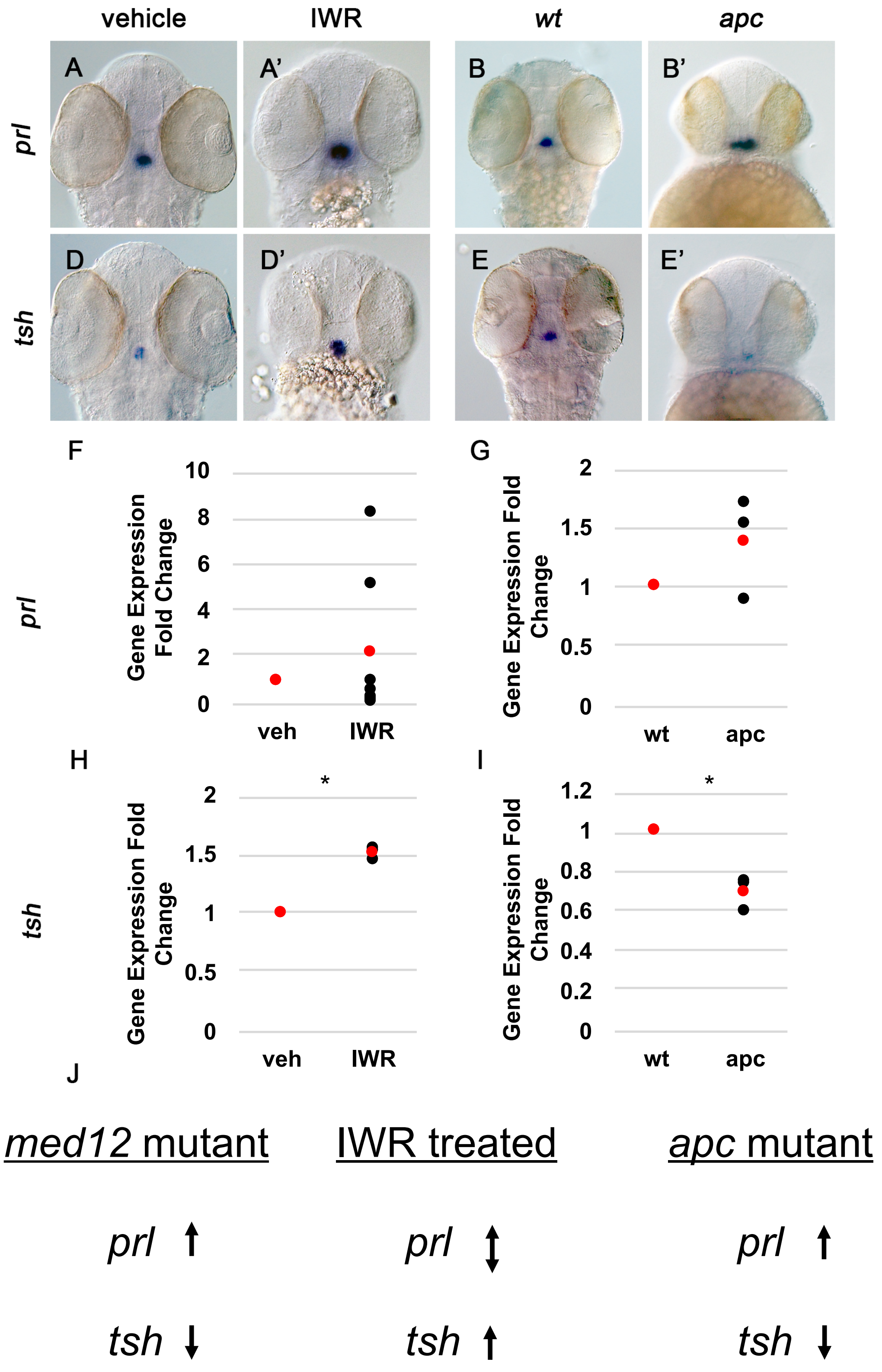

Biology Open • Supplementary information

Figure S1. Alterations in Wnt signaling modulate *prl* and *tsh* gene expression in the pituitary. (A, A’, B, B’, D, D’, E, E’) are all dorsal views of WISH in 48 hpf embryos magnified x100. (A, D) *wt* siblings, vehicle treated. (A’, D’) *wt*, IWR-treated embryos. (B, E) *wt* siblings. (B’, E’) *apc* mutant embryos. (A, A’, B, B’) *prl* expression in the pituitary. (D, D’, E, E’) *tsh* expression in the pituitary. Expression of *prl* (F, H) and *tsh* (G, I) were measured in IWR-treated (F, H) and *apc* mutant (G, I) 48 hpf embryos using RT-qPCR. Gene expression values were normalized to an endogenous control, *beta actin 2* (*actb2*), and are expressed as fold changes relative to *wt* sibling, or vehicle-treated *wt* sibling controls. Dot plots show three biological replicates (black) and the mean (red). (J) Schematic comparing *prl* and *tsh* gene expression changes in *med12* mutant embryos, IWR-treated embryos, and *apc* mutant embryos.

Biology Open • Supplementary information

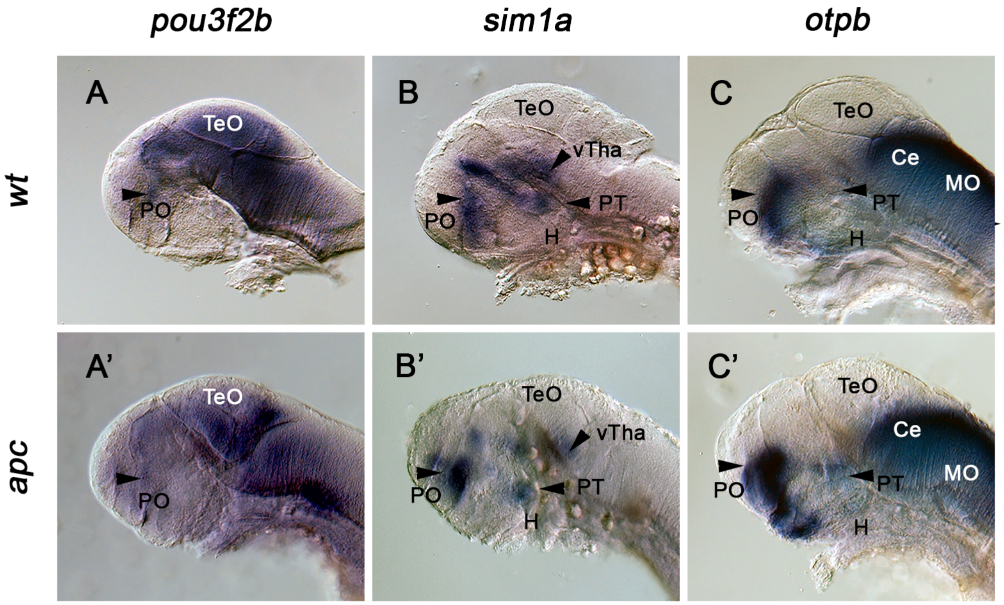

Figure S2. *Pou3f2b, sim1a,* and *otp* are expressed in the pre-optic area of the *apc* mutant. (A, A', B, B', C, C') are all lateral views of WISH in 48 hpf embryos magnified x100 in which arrowheads indicate the PO. (A, B, C) wild type siblings. (A'. B', C') *apc* mutant embryos. (A, A') *pou3f2* expression in the PO. (B, B') *sim1a* expression in the PO, PT and vTha. (C, C') *otp* expression in the PO, PT, and H. PO, pre-optic area; vTha, ventral thalamus; PT, posterior tuberculum; H, hypothalamus; TeO, optic tectum; Ce, cerebellum; MO, medulla oblongata.

Table S1. Lethality of LiCl treatment differs depending on developmental stage.

**Stage treated**	**Duration**	**Percent surviving treatment**
5 hpf	20 minutes	55 %
6 hpf	12 hours	0 %
8 hpf	30 minutes	83.3 %
9 hpf	2 hours	0 %
10 hpf	1 hour	100%
11 hpf	30 minutes	100%
12 hpf	18 hours	0 %
18 hpf	3 hours	100%
20 hpf	1 hour	100 %
24 hpf	24 hours	0 %
24 hpf	1 hour	100 %
30 hpf	1 hour	100 %
31 hpf	4.5 hours	100%
41 hpf	5 hours	100%
